# Antineutrophil cytoplasmic antibody-associated vasculitis: insights into relapse risk and future management directions

**DOI:** 10.3389/fimmu.2025.1655326

**Published:** 2025-09-18

**Authors:** Federico Alberici, Oliver Flossmann, Peter Lamprecht, Kevin W. Loudon, Roberto Padoan, Tamara Popov, Carlo Salvarani, Aladdin J. Mohammad

**Affiliations:** ^1^ Nephrology Unit, University of Brescia, ASST Spedali Civili, Brescia, Italy; ^2^ Renal Unit, Royal Berkshire Hospital, Reading, Berkshire, United Kingdom; ^3^ Department of Rheumatology and Clinical Immunology, University of Lübeck, Lübeck, Germany; ^4^ Molecular Immunity Unit, Department of Medicine, University of Cambridge, Cambridge, United Kingdom; ^5^ Cambridge University Hospitals NHS Foundation Trust, NIHR Cambridge Biomedical Research Centre, Cambridge, United Kingdom; ^6^ Rheumatology Unit, Department of Medicine (DiMed), University of Padua, Padua, Italy; ^7^ CSL Vifor, Glattbrugg, Switzerland; ^8^ Rheumatology Unit, Azienda USL-IRCCS di Reggio Emilia, Università di Modena e Reggio Emilia, Reggio Emilia, Italy; ^9^ Department of Rheumatology, Skåne University Hospital, Lund, Sweden; ^10^ Clinical Sciences, Rheumatology, Lund University, Lund, Sweden; ^11^ Department of Medicine, University of Cambridge, Cambridge, United Kingdom

**Keywords:** antineutrophil cytoplasmic antibody (ANCA)-associated vasculitis, AAV relapse, granulomatosis with polyangiitis (GPA), microscopic polyangiitis (MPA), remission re-induction

## Abstract

Antineutrophil cytoplasmic antibody (ANCA)-associated vasculitis (AAV) has a relapsing-remitting course and, even with the availability of effective maintenance therapies such as rituximab, relapse rates remain high. Relapse is associated with the accrual of organ damage stemming from both the underlying disease and from the effects of AAV treatments; thus, early detection and proactive prevention are crucial. AAV study populations typically include mixed cohorts of patients with new-onset and relapsing disease. Although data specifically addressing re-induction of remission after relapse are limited, available evidence suggests high remission rates when rituximab is combined with glucocorticoids. However, the balance between effective disease control and the potential treatment-related side effects must be carefully considered, and new therapeutic options may help improve this tradeoff. The aim of this review is to explore what is known about relapse risk and relapse management while considering emerging pathogenic and therapeutic paradigms.

## 1 Introduction

Antineutrophil cytoplasmic antibody (ANCA)-associated vasculitis (AAV) is a group of autoimmune necrotizing small vessel vasculitides that causes potentially life-threatening ischemic and inflammatory organ damage (1–3). The three subsets of AAV are granulomatosis with polyangiitis (GPA), microscopic polyangiitis (MPA), and eosinophilic granulomatosis with polyangiitis (EGPA). Patients with GPA predominantly have ANCA directed against proteinase 3 (PR3), whereas, in MPA, 60 – 80% of patients have ANCA specific for myeloperoxidase (MPO) (4, 5). In EGPA, MPO-ANCA are detected in 30 – 40% of patients, whereas PR3-ANCA are rare. GPA is the most common AAV subtype with a global incidence of 9 per million person-years (6). Due to the distinct nature and genetic background of EGPA, it is often excluded from AAV clinical trials and will not be discussed in this paper.

Treatment of AAV with cytotoxic agents, immunosuppressive therapies, and biologics (particularly the monoclonal anti-CD20 antibody rituximab) have improved prognosis by effectively addressing active disease which, if untreated, can lead to impaired organ structure and/or function (organ damage) (7, 8). Although AAV is associated with premature mortality relative to the general population (9, 10), treatment-related improvements in survival together with a greater awareness of AAV leading to improved diagnosis have led to a rise in the overall prevalence of AAV (6, 11, 12).

These conditions, which were once fatal in nearly all patients, are now considered as chronic relapsing disorders with peaks and troughs in disease activity (13). Although relapses (defined as the return of active disease after remission) are common and can be clinically significant, it is not possible to reliably predict when they will occur. Re-establishing control of disease activity (re-induction) promptly in patients with relapse is important to prevent or minimize organ damage. However, the optimal treatment for AAV relapse remains to be determined (14).

This paper summarizes the risk factors for AAV relapse, evaluates the effectiveness of available and exploratory biomarkers for predicting relapse, explores factors that might help inform relapse prevention strategies, and assesses the available treatment options for re-inducing remission.

## 2 Pathophysiology of AAV

Immune dysfunction is fundamental to the development of characteristic inflammatory lesions in blood vessels and affected organs in AAV (2, 3). In patients with impaired immunological self-tolerance and a genetic predisposition, the production of ANCA plays a key role in AAV pathogenesis by promoting inflammatory responses (4, 15, 16). The binding of ANCA to MPO and PR3 exposed on neutrophils primed by complement fragment C5a and cytokines enhances leukocyte-endothelial interactions triggering neutrophil extracellular trap (NET) formation (17) as well as a strong inflammatory response. This process is part of a broader immune dysfunction which exacerbates endothelial injury, with C5a further amplifying inflammation by enhancing the generation of antibodies and the activation of phagocytic cells (18). Complement activation thus plays a key role in disease pathogenesis and therefore in the development of organ injury (19–22). Of note, pathophysiological studies in AAV have historically not distinguished between the mechanisms driving disease onset and those involved in relapse. Although these processes are likely to overlap, definitive evidence supporting this assumption is currently lacking.

## 3 AAV disease course

**Figure 1:**
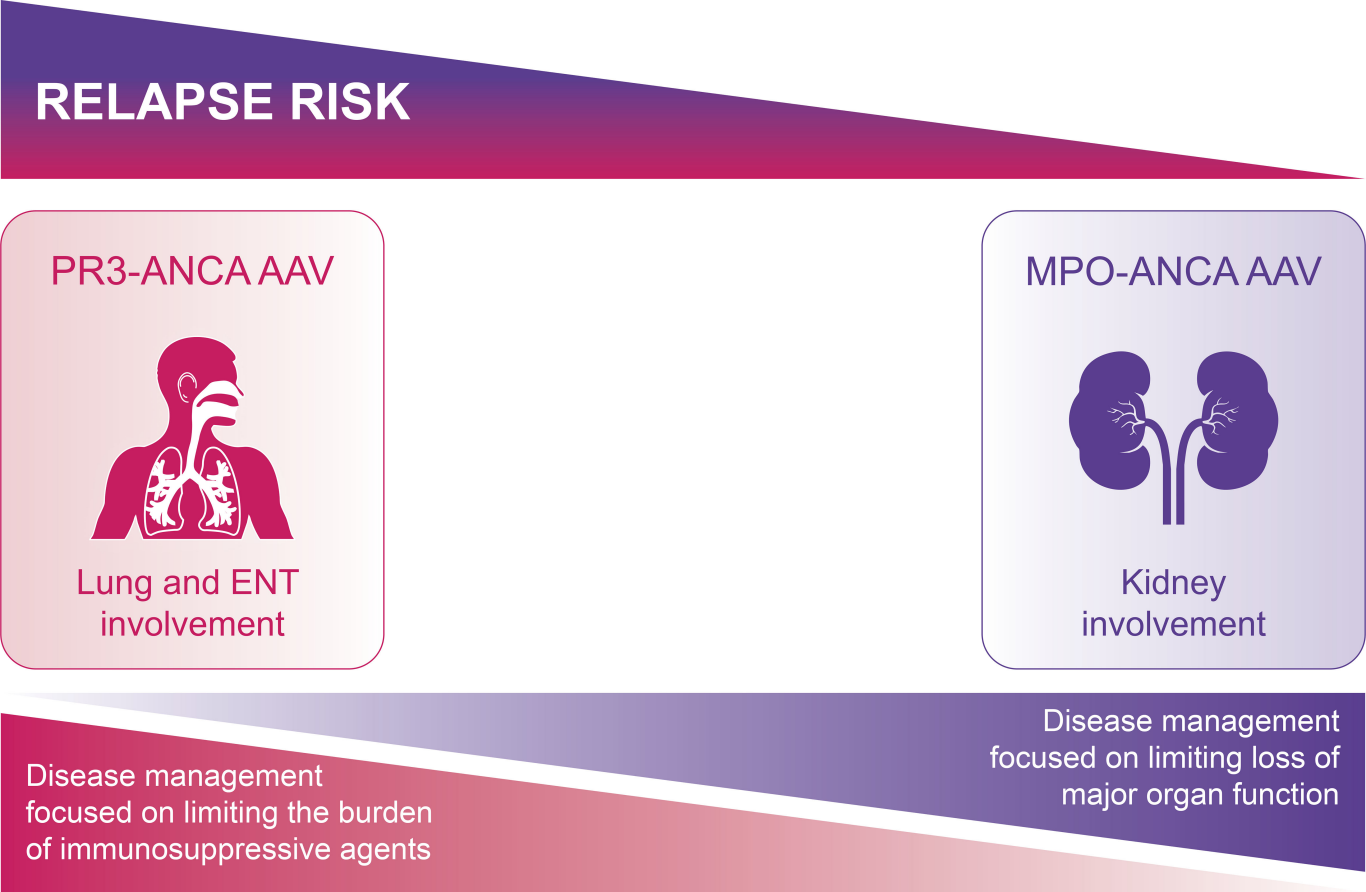
Aims of treatment differ according to AAV phenotype. For PR3-ANCA AAV, the relapse rate is higher, but the risk of major organ damage is lower, and so limiting the burden of re-treatment is the main aim of disease management. For MPO-ANCA AAV, although the relapse risk is lower, the higher chance of residual organ damage from the first relapse means there is higher risk of further progression of organ damage towards end-stage disease; the aim of disease management is thus to limit disease activity and reduce the risk of further loss of major organ function. AAV, ANCA-associated vasculitis; ANCA, antineutrophil cytoplasmic antibody; MPO, myeloperoxidase; PR3, proteinase 3.

AAV can develop at any time, but incidence increases with age (23). This complex disease has heterogenous phenotypes resulting in different treatment priorities in different patients (**Figure 1**).

The early stages of AAV typically present with a range of non-specific symptoms suggestive of chronic inflammation, including fatigue, weight loss, fever, myalgia, and polyarthralgia (23). Patients with more advanced disease may show signs and symptoms of damage to the ears, nose and throat (ENT) (e.g., chronic rhinosinusitis, nasal bleeding and hearing loss (23, 24)), lung fibrosis (e.g., persistent cough and breathlessness (23)), and/or chronic kidney disease (CKD) (e.g., microscopic hematuria and reduced estimated glomerular filtration rate [eGFR] (23)). In addition, prolonged active disease can result in inflammation-related comorbidities, such as cardiovascular disease (25, 26). The longer AAV remains undiagnosed and untreated, the greater the risk of AAV-related comorbidities and irreversible organ damage.

AAV is a relapsing-remitting disease making increases in disease activity part of the natural disease course. However, there is considerable variability in the duration of remission between relapses (27, 28), with some patients never experiencing a relapse. These disparities may be partly explained by differences between patients in immune system characteristics and response to immunosuppressive treatment (29, 30).

Each relapse increases the risk of potentially fatal organ damage due to increased inflammatory disease activity and the use of higher-dose (more toxic) re-induction treatments (31). Mortality is also affected by treatment-related toxicities (in particular serious infections) and comorbidities, including cardiovascular disease (26). Although treatment effectively improves survival in most patients, survival rates are typically lower in older patients and in those with more severe kidney involvement and more active disease (32). Despite improvements in survival rates over recent decades, mortality remains approximately 2.7 times higher in patients with AAV than in the general population (26).

## 4 Treatment of AAV

The aim of AAV treatment is to control active disease whilst limiting treatment-related toxicities, thereby preventing or minimizing the risk of tissue damage. Treatment includes intensive induction therapy (typically high-dose glucocorticoids with rituximab or cyclophosphamide) to gain rapid disease control and, once this is achieved, less toxic maintenance strategies to sustain remission (14).

The choice of treatment is not straightforward and may be restricted by the presence of disease-induced organ damage and co-morbidities. Treatments should ideally be tailored according to disease activity, risk factors, age, and existing comorbidities, e.g., CKD and lung disease (14, 33). The risk of treatment-induced comorbidities, such as infertility, diabetes, osteoporosis and infections, must also be considered. Ongoing monitoring of risk factors and signs of relapse is recommended throughout remission, as relapse risk can fluctuate over time due to inherent factors and cumulative events. The ideal scenario would be to have reliable tools for predicting relapses so, where necessary, treatment can be intensified early.

Over the last 30 years, expanding AAV treatment options have led to a shift from high-dose glucocorticoids supplemented with cyclophosphamide to less toxic approaches (34–36). For example, cyclophosphamide is often replaced or supplemented with rituximab to reduce the cumulative dose (14, 33). The current consensus is that rituximab is the most effective treatment for maintaining remission (37–39), and a combination of rituximab and glucocorticoids is recommended for the treatment of relapsing disease in most settings (14, 33). According to treatment guidelines, once remission is established, glucocorticoids should be tapered to the lowest effective dose (typically ≤5 mg/day within 4 to 5 months) or completely withdrawn to limit treatment toxicity (14, 33).

Recent studies, including the phase 3 randomized controlled ADVOCATE trial (40), demonstrate that the complement 5a receptor 1 (C5aR1) antagonist, avacopan, can improve remission rates, sustain remission over time, and (in patients with ANCA-associated glomerulonephritis) improve kidney function in patients with new-onset or relapsing AAV treated with rituximab or cyclophosphamide and reduced glucocorticoid exposure (37, 39–44). The use of avacopan with a low-dose glucocorticoid regimen is associated with a lower incidence of toxicities, including serious infections, than standard (non-avacopan) treatment (45). Based on results from the ADVOCATE trial (40), EULAR and KDIGO guidelines recommend using avacopan to reduce glucocorticoid exposure in patients receiving standard treatment for GPA or MPA (14, 33).

## 5 Challenges in maintaining remission

The optimal maintenance regimen has yet to be defined for patients achieving remission. Based on results from the MAINRITSAN trial (**Table 1**) (39), the recommended regimen includes pre-emptive rituximab doses of 500 mg every six months for 24 months (14, 53). However, the RITAZAREM trial found that a rituximab-based regimen of 1000 mg every 4 months was also effective in patients with relapsing disease (51) leading guidelines to suggest using the higher dose in patients who relapse on the 500 mg regimen (14).

**Table 1:** Summary of key data from randomized clinical trials in patients with relapsing AAV.

Trial	Study treatment	Follow-up	Remission assessment	Relapse assessment	Incidence of infection
Rituximab					
RAVE (37, 46) N=188 • Around half of patients had relapsing AAV • Excluded patients with severe kidney involvement	Arm 1: RTX 375 mg/m^2^/week×4 (n=99). No active treatment after achieving remission (n=61) to Month 18 Arm 2: Daily CYC 2 mg/kg (n=98). On achieving remission (n=63) AZA 2 mg/kg to Month 18	18 months	Rate of remission at 6 months in relapsing AAV: RTX 67% p=0.01 CYC/AZA 42%.	Number of major relapses at: 12 months • 7 RTX group • 15 CYC/AZA group p=0.03 18 months • 1 RTX group • 17 CYC/AZA group p=0.19	Rate of grade ≥3 infections similar for both treatment groups (12% vs 11%) Leukopenia grade ≥2 was less common in RTX group (5 vs 23%, P<0.001)
RAVE re-induction (47) N=26 (15 RTX; 11 CYC/AZA • Patients from RAVE who experienced severe relapse Months 6 – 18 • 16 patients had relapsing AAV on starting RAVE	Open-label RTX (375 mg/m^2^/week for four weeks) plus GC (1mg/kg, tapered to discontinuation during remission)	12 months	Remission restored with RTX and GC re-induction treatment in 88% of patients 13/15 RTX group 10/11 CYC/AZA group Complete remission (zero GC) 40% RTX group 64% CYC/AZA group Mean time to remission: 56 days RTX group 36 days CYC/AZA group	Relapse rate at 12 months: • 2 limited relapses RTX group • 2 severe relapse CYC/AZA group	13 infections, of which 10 (77%) involved the ears, nose and upper respiratory tract. 2 grade 3 infections (gastroenteritis and sinusitis)
MAINRITSAN-1 (39) N=115 • Patients with AAV in remission • 20% had relapsing disease	Arm 1: Fixed RTX 500 mg infusion every 6 months for 18 months (n=57) Arm 2: Azathioprine 1 – 2 mg/kg/day for 22 months (n=58)	28 months	Prior to enrolment, remission was achieved using CYC plus GC and obtained after a mean of 4.6 months	Rate of major relapse Month 28: • 5% RTX group • 29% AZA group p= 0.002 4 AZA patients switched to RTX. In the AZA group 8 major relapses occurred within the first 12 months of maintenance therapy. One major relapse in the RTX group occurred at Month 8, and the 2 others occurred after the last infusion (Month 22 and Month 24)	Severe infections: • 19% RTX group • 14% AZA group Fatal sepsis in 1 AZA patient
MAINRITSAN-1 follow-up (48) N=115 • Patients with AAV in remission • 20% had relapsing disease	Arm 1: Fixed RTX 500 mg infusion every 6 months for 18 months (n=57) Arm 2: Azathioprine 1 – 2 mg/kg/day for 22 months (n=58)	60 months		At month 60, the major relapse-free survival rates were: 71.9% RTX group 49.4% AZA group p=0.003 RTX patients had 12.6 months more without relapse or toxicity than AZA patients	Severe infections: • 26% RTX group • 28% AZA group More bronchitis events with RTX v AZA (10 vs 1)
MAINRITSAN-2 (49) N=115 • Patients with AAV in remission • Tailored group 35% relapsing AAV • Fixed group 37% relapsing AAV	Arm 1: RTX 500 mg infusion + further RTX reinfusion when CD19+B lymphocytes reappeared or ANCA titre rose markedly (tailored group; n=81) Arm 2: Fixed RTX 500 mg infusion on days 0 and 14, then 6, 12 and 18 months after the first infusion (Fixed group; n=81)	28 months		Withdrawal due to major relapse by Month 18: • Tailored group: 3 • Fixed group: 2 Relapse rate Month 28: • Tailored: 17.3% • Fixed: 9.9% p=0.22 Major relapse rate Month 28: • Tailored: 83.8% • Fixed: 86.4% p=0.23	Each group had 18 infections. Infectious complications: • 11% tailored RTX • 20% fixed RTX group
MAINRITSAN-3 (50) • 68 patients in complete remission from MAINRITSAN - 2 • 41% relapsing disease	Arm 1: RTX every 6 months for 18 months (4 infusions) (n=50) Arm 2: Placebo infusion every 6 months for 18 months (4 infusions) (n=47)	≤36 months		Relapse-free survival Month 28 Any: • RTX: 96% • Placebo: 74% p=0.008 Major: • RTX: 100% • Placebo: 87% p=0.009 Month 48 relapse rates Any: • RTX: 4% • Placebo: 26% Major: • RTX: 0% • Placebo: 13%	Incidence of serious infection • 12% RTX group • 9% placebo group
MAINRITSAN Pooled (8) N=277 • 29% relapsing AAV	Group 1: 18-month fixed-dosing RTX (n=97) Group 2: 18-month tailored RTX (n=40) Group 3: 36-month tailored/fixed RTX (n=42) Group 4: 36-month fixed/fixed RTX (n=41) Group 5: AZA (n=58)	84 months		Major relapse risk Month 84: 18-month fixed RTX superior to AZA (HR 0.38) 18-month fixed RTX superior to tailored RTX (HR 2.92) Similar for 36-month fixed/fixed RTX and 18-month fixed RTX (HR 0.69) Median time to major relapse was 25 months with AZA group, and 36 months with RTX	Infections were the most frequent serious adverse event with 75 (27%) patients having ≥1 serious infection
RITAZAREM (51, 52) N=188 • Patients with relapsing AAV recruited at time of relapse • 63% of relapses had ≥1 major disease activity item	Re-induction with weekly RTX and GCs (N = 187) followed by: Arm 1: maintenance with fixed-dose RTX 1000 mg every 4 months, through month 20 (n=85) Arm 2: Daily AZA for 24 months (n=85)	48 months	Re-induction of remission was successful within 4 months in 90% of patients Of the 17 patients who did not achieve remission by Month 4, 13 (76%) had PR3-ANCA AAV and 10 (59%) had ENT involvement at baseline	RTX was superior to AZA in preventing any relapse (HR 0.41; p<0.001) and major relapse was (HR 0.36; p=0.004). 38 RTX patients (45%) had 52 relapses (11 major). 60 AZA patients (71%) had 89 relapses (28 major).	Induction phase 5/13 severe infections occurred within 4 weeks of the first RTX induction dose. There were 86 non-severe infections 2 patients died in the induction phase due to pneumonia Maintenance phase 19 serious infections in the RTX group and 24 in the AZA group.
Avacopan					
Phase II trial (43) N=67 Relapsing AAV: • GC group: 22% • GC+AVA group: 32% • AVA group: 27%	CYC or RTX plus GC 60 mg/day or GC 20 mg/day and AVA 30 mg bid or AVA 30 mg bid	12 weeks	Remission at Week 12: • GC group: 40% • GC+AVA group: 45% • AVA group: 33%	Remission at Week 4 sustained to Week 12: • GC group: 5% • GC+AVA group: 14% • AVA group: 29% (p<0.05 vs GC group)	Serious infection: • GC group: 4% • GC+AVA group: 5% • AVA group: 5%
ADVOCATE (40) N=331 Relapsing AAV: • GC group: 30.5% • AVA group: 30.7%	AVA 30 mg bid (n=166) or Tapered prednisone (n=165) Along with RTX as single cycle at the time of induction or CYC followed by AZA	52 weeks	AVA was non-inferior to prednisone in achieving remission at Week 26 (72.3% vs 70.1%; p<0.0001)	AVA was superior to prednisone in maintaining remission at Week 52 (65.7% vs 54.9%; p=0.0066) The risk of relapse at 52 weeks was 54% lower with AVA vs prednisone	Serious infection: • AVA group: 13.3% • GC group: 15.2% Death due to infection: • AVA group: 0.6% • GC group: 1.2%

AZA, azathioprine; AVA, avacopan; CYC, cyclophosphamide; ENT, ear, nose and throat; GC, glucocorticoid; HR, hazard ratio; RTX, rituximab.

Defining the optimal duration of rituximab treatment is particularly challenging due to the increased risk of adverse effects, including infection, hypogammaglobulinemia, and impaired vaccine response. The recommended duration of rituximab therapy varies between guidelines, with EULAR guidelines recommending continuing treatment for 24 to 48 months (14) and KDIGO guidelines recommending 18 to 48 months (33). Of note, the MAINRITSAN-3 trial demonstrated fewer relapses in patients receiving rituximab for 36 months versus 18 months (50). Although subsequent pooled analyses failed to show an improvement in relapse-free survival with extended dosing (8), results suggest that extending maintenance therapy may be beneficial in some contexts (54).

Rituximab maintenance treatment is generally effective in sustaining remission and is typically well-tolerated. However, this is not the case for all patients and several factors may limit its’ use. These include a reduced response to rituximab in some patients, which may be a consequence of high interpatient variability of serum rituximab levels due to genetic polymorphisms and/or (in patients with repeated rituximab exposure) neutralization of rituximab activity by anti-rituximab antibodies (55–57). Furthermore, the use of rituximab is associated with an increased risk of infection, as demonstrated by both the MAINRITSAN and RITAZAREM trials (**Table 1**) (39, 52). The observation that infection rates improved using an individually tailored approach with reduced rituximab exposure (49) raises the question of whether decisions abouts re-dosing and the duration of maintenance treatment should always be pre-emptive or whether they should be reduced in people with low relapse risk (e.g., those who remain MPO-ANCA negative or experience sustained B-cell depletion after rituximab treatment) (**Table 2**) (62, 63).

**Table 2:** Risk factors for AAV relapse.

Factor	Associated relapse risk	Additional remarks
Patient clinical characteristics		
Older age (58)	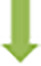	Patients with AAV after the age of 75 years have a lower relapse risk than patients aged 65 – 75 years
Molecular characteristics		
HLA-DPB1*04:01 (59)	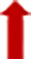	Homozygosity for this allele, which is mainly found in PR3-ANCA positive disease, is associated with a higher relapse risk
Leukocyte PRTN3 expression (60)	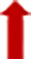	Mainly found in PR3-ANCA positive disease
Low levels of endogenous anti-C5aR1 antibodies (22)	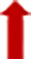	Good correlation with disease activity
Baseline disease characteristics		
MPO-ANCA positive (27, 61)	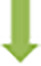	
PR3-ANCA positive (27, 59–61)	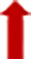	
Disease characteristics during treatment		
Persistently negligible ANCA levels (for MPO-ANCA positive patients) (62)	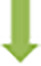	Patients who remain ANCA negative were found to remain relapse-free
Persistent B-cells depletion (63)	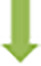	Patients who remain sustained B-cell depleted after rituximab were found to remain relapse-free
Serological remission (64)	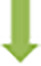	Achieving serological remission (negative ANCA assay) within 180 days of induction was associated with a lower 5-year cumulative incidence of relapse (9.4 per 100 patients vs 18.3)
History of relapse (27)	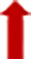	Once a patient has experienced a relapse, they are more likely to do so again
Increasing ANCA concentrations (65)	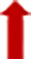	A two-fold increase in ANCA levels was found to be associated with a significantly increased relapse risk
Seroconversion from negative to positive ANCA (62, 64, 66, 67)	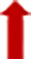	Several trials have reported that a change from negative to positive ANCA titre is associated with increased relapse risk
ANCA positivity (66, 67)	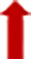	Persistent ANCA positivity, despite being in remission is associated with an increased relapse risk
Increase in peripheral B-cells (63, 68)	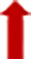	The return of peripheral B-cells following rituximab-induced B cell depletion is associated with an increased risk of relapse
Maintenance treatment employed		
Rituximab based maintenance treatment (39, 52)	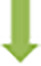	Compared to the historical standard of care (azathioprine)
Morbidities		
End-stage kidney disease (69)	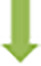	End-stage kidney disease is generally associated with a low risk of relapse, but it can increase the risk of infection that raises relapse rate
Infection (70, 71)	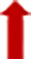	Infection can promote AAV activity, increasing the risk of relapses

AAV, ANCA-associated vasculitis; ANCA, antineutrophil cytoplasmic antibody; MPO, myeloperoxidase; PR3, proteinase 3.

The risk of infection tends to increase in patients with hypogammaglobulinemia during the first year of rituximab induction. This is more prevalent among patients with low baseline IgG levels and is primarily linked to older age and glucocorticoid dose (72–75). Although hypogammaglobulinemia is not definitively linked to an increased risk of infection during maintenance treatment, patients receiving re-induction therapy after relapse may experience further decreases in IgG levels due to repeated rituximab administration (75, 76). Similarly, the diminished response to vaccines in patients undergoing B-cell depletion—a common phenomenon in rituximab-treated patients —may have important clinical implications, potentially heightening the risk of infection (77, 78).

The high risk of severe infections and other complications with glucocorticoids, even at low doses, means their use requires careful risk-benefit assessment, particularly in terms of co-morbidities (31). Whilst pre-rituximab data suggest that the extended use of glucocorticoids is associated with fewer relapses (79), a retrospective study in rituximab-treated patients found that extending the use of high-dose glucocorticoids for more than six months increased the risk of severe infection and other adverse effects (e.g., diabetes and cardiovascular complications) without reducing relapse risk (80). The long-term use of low-dose glucocorticoids for the prevention of relapse has not been specifically evaluated in randomized controlled trials. However, the TAPIR trial found that the risk of major relapse in rituximab-treated patients with GPA remained low irrespective of whether patients received low-dose or no glucocorticoids and that the benefits of low-dose glucocorticoids to prevent minor relapses were only observed among patients treated with non-rituximab-based regimens (81). This suggests that maintenance treatment with rituximab and little or no glucocorticoids may be possible in some patients.

## 6 Relapse in AAV

Relapse is classified according to the level of disease activity and the extent of organ involvement, assessed using the Birmingham Vasculitis Activity Score (BVAS) Version 3 (14, 82) or the Birmingham Vasculitis Activity Score for Wegener’s Granulomatosis (BVAS-WG) (83). A relapse is typically deemed major if it affects key organs, such as the neurological system or the kidneys. Minor relapses are usually characterized by constitutional symptoms either in isolation or in association with non-life or non-organ-threatening manifestations. However, classification is often complicated by limitations in assessing disease activity. For example, kidney disease relapse does not have a generally accepted and shared definition, and there is no standardized modality for assessing kidney disease activity in patients with relapsing AAV.

ANCA testing is a central component of AAV diagnosis and is increasingly used to help define disease status, including remission and relapse. A negative serum ANCA assay characterizes serological remission while ANCA return suggests serological relapse. However, since ANCA titers do not necessarily reflect AAV activity, they are an imperfect indicator of relapse (78).

In the pre-rituximab era, the likelihood of relapse within the first five years was 40%–55% (82, 84). While the use of rituximab as maintenance therapy has significantly reduced this risk, relapse rates are not negligible and tend to increase significantly after maintenance treatment is withdrawn (8).

### 6.1 Relapse risk

Accurate risk assessment is essential for the prevention or early detection of relapse (31). Consequently, guidelines recommend continuously assessing patients throughout their journey (14, 33). Several factors determine the likelihood of relapse (**Table 2**). Generally, relapse is less common in patients with MPA (27), MPO-ANCA associated disease (27, 61), severe kidney disease (69), and older age (>75 years old) (58), while it is more common in those with GPA (27), PR3-ANCA associated disease (27, 59–61), and ENT involvement (66, 68, 70, 85–87). Other risk factors for relapse include infection (70, 71), relapse history (27), seroconversion to ANCA positivity and (possibly) rising ANCA titers during treatment (62, 64–67) (**Table 2**). In contrast, persistent ANCA negativity (especially in rituximab-treated patients with sustained B-cell depletion) is strongly predictive of a patient remaining relapse-free (62, 63, 68).

When assessing relapse risk in patients with AAV, the therapeutic regimen employed should also be taken into account. While robust evidence has established that a rituximab-based maintenance regimen is superior to azathioprine in preventing relapses (39, 52), emerging data suggest that the induction regimen may also influence relapse risk. A retrospective real-world study involving 101 patients with AAV demonstrated that an induction strategy combining rituximab and cyclophosphamide was associated with a lower relapse rate compared to rituximab monotherapy (88). Notably, this association was observed only when both major and minor relapses were considered together and was not confirmed for major relapses alone. Further evidence for the impact of combined induction with rituximab and cyclophosphamide compared to rituximab alone will be provided by the ongoing, randomized controlled ENDURRANCE trial (89).

The underlying mechanisms driving relapse are not fully understood; consequently, our understanding of the relevance of concomitant risk-modifying factors is limited. ENT damage, for example, is associated with a higher relapse risk but a lower risk of kidney disease and better survival rates (87). The high number of relapse risk factors ranging from inherent factors (e.g., ANCA specificity) to single patient “disease behavior” and comorbidities (e.g., previous relapse and infection) suggests a need for advanced modelling techniques that provide accurate estimates of future relapse risk.

### 6.2 The role of biomarkers to detect imminent relapse

Although ANCA levels, the timing of B-cell return after rituximab treatment, and (in some contexts) complement fluctuations may provide some indication of impending relapse (22, 62, 65, 68), the search for a reliable and reproducible biomarker continues (90). In addition to predicting relapse, biomarkers may provide information on factors that impact overall prognosis (e.g., subclinical disease activity) and help identify patients with an abnormal activation of fibrotic pathways that may contribute to organ damage (91). As our understating of AAV expands and reports emerge of several disease phenotypes (92), it is becoming increasingly likely that a reliable biomarker for patients with one phenotype may not necessarily be appropriate for patients with other characteristics.

#### 6.2.1 Clinical biomarkers

Although increases in ANCA titers are typically associated with increased relapse risk, data from clinical trials and observational studies are often conflicting (8, 46, 49). In patients treated with rituximab, B-cell repopulation after complete peripheral depletion may help inform the risk of relapse and the optimal timing of rituximab administration within a tailored retreatment strategy (63, 66). This hypothesis has been tested in a prospective study, in which B-cell-driven rituximab maintenance therapy was more effective than ANCA-driven rituximab therapy for preventing relapse (78). Therefore, although the peripheral B-cell count monitored for personalized treatment does not necessarily correlate with tissue-resident B-cells, it may be a useful clinical indicator for relapse (93). Of note, characteristics of the B-cell compartment show significant interpatient variability at the time of repopulation and may provide an even more accurate indication of relapse risk (94). Indeed, a greater relapse risk was reported for repopulating B-cell compartments comprising a higher proportion of autoreactive PR3+ B-cells, switched memory B-cells or plasmablasts, and a lower proportion of naïve B-cells (94–96). This suggests that combining ANCA and B-cell monitoring (at least in rituximab-treated patients) may provide useful information on relapse risk in patients with a relative low relapse rate, such as those with MPO-ANCA MPA who exhibit persistent ANCA negativity and B-cell depletion.

Eventually, B-cell repopulation is likely to occur in rituximab-treated patients and further research should focus on determining how to optimize the depletion of the B-cells subsets that drive autoimmunity. It should also be noted that relapses may occur in patients without B-cell repopulation or increasing ANCA titers (48, 63, 66), and that some biomarkers may provide useful information on relapse risk in specific organs.

#### 6.2.2 Exploratory biomarkers

Although exploratory biomarkers are not yet ready for use in clinical practice, they may provide useful insights into disease pathogenesis. Some of these biomarkers have been tested in randomized controlled studies (43).

A retrospective analysis of samples collected during the RAVE trial found high levels of interleukin-6 (IL-6) that positively correlated with ANCA levels in patients with PR3-ANCA but not MPO-ANCA (28).

In addition to IL-6, low levels of autoantibodies targeting C5aR1 show good correlation with both AAV disease activity and relapse risk (22). This suggests a physiologically antagonistic role for endogenous anti-C5aR1 antibodies as regulators of a C5aR1 immune checkpoint and provides support for the use of therapies such as avacopan, which target the complement system in patients with AAV.

Calprotectin is released by neutrophils and monocytes during inflammation and correlates with active AAV (97, 98). Increases in serum calprotectin may predict AAV relapse as a potential indicator of sub-clinical kidney inflammation (99, 100).

The potential role of NETs as biomarkers is supported by their central role in the pathogenesis of AAV (17). Notably, neutrophils expressing type II interferon (IFN) signature genes are increased in patients with MPA and are associated with persistent vasculitis symptoms (101). Furthermore, elevated IFN-γ levels at disease onset, a key cytokine driving the differentiation of mature neutrophils toward a type II IFN signature phenotype, are associated with an increased risk of disease relapse (101). These findings, derived from a Japanese cohort, warrant validation in other ethnic populations to assess their generalizability.

Finally, urinary biomarkers may inform on the status of kidney disease activity. MCP1 and CD163 have been associated with kidney vasculitis relapses (102, 103) and increases in urinary CD4+ T-cell (104, 105). Among the exploratory biomarkers, MCP-1 and CD163 appear to be the most advanced in terms of potential clinical application (40).

### 6.3 Treatment of relapse

**Figure 2:**
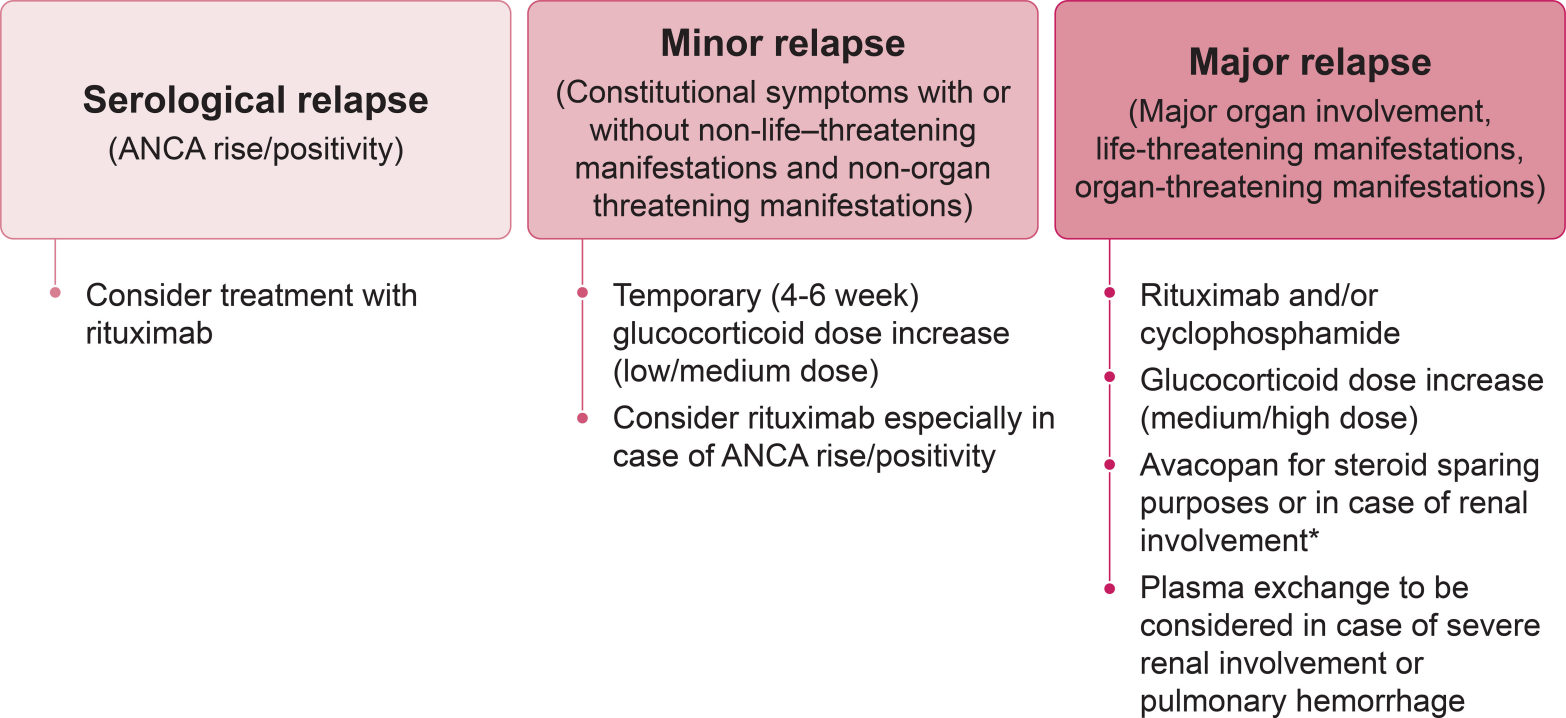
Flow-chart outlining the recommended approach for managing AAV relapse. * If avacopan is started, reduce/withdraw glucocorticoids in 4 – 6 weeks. ANCA, antineutrophil cytoplasmic antibody.

Glucocorticoids are frequently included in treatment strategies for relapse with the dose varying according to relapse severity and treating physician experience (**Figure 2**). The risk of glucocorticoid-related toxicity is potentially higher in patients with previous relapses due to prior glucocorticoid exposure. The ideal form of management is therefore prevention and, since relapse frequently occurs after the cessation of maintenance therapy, extending the duration of maintenance treatment for patients with known risk factors (**Table 2**) could help reduce the likelihood of relapse (52, 66, 106).

**Figure 3:**
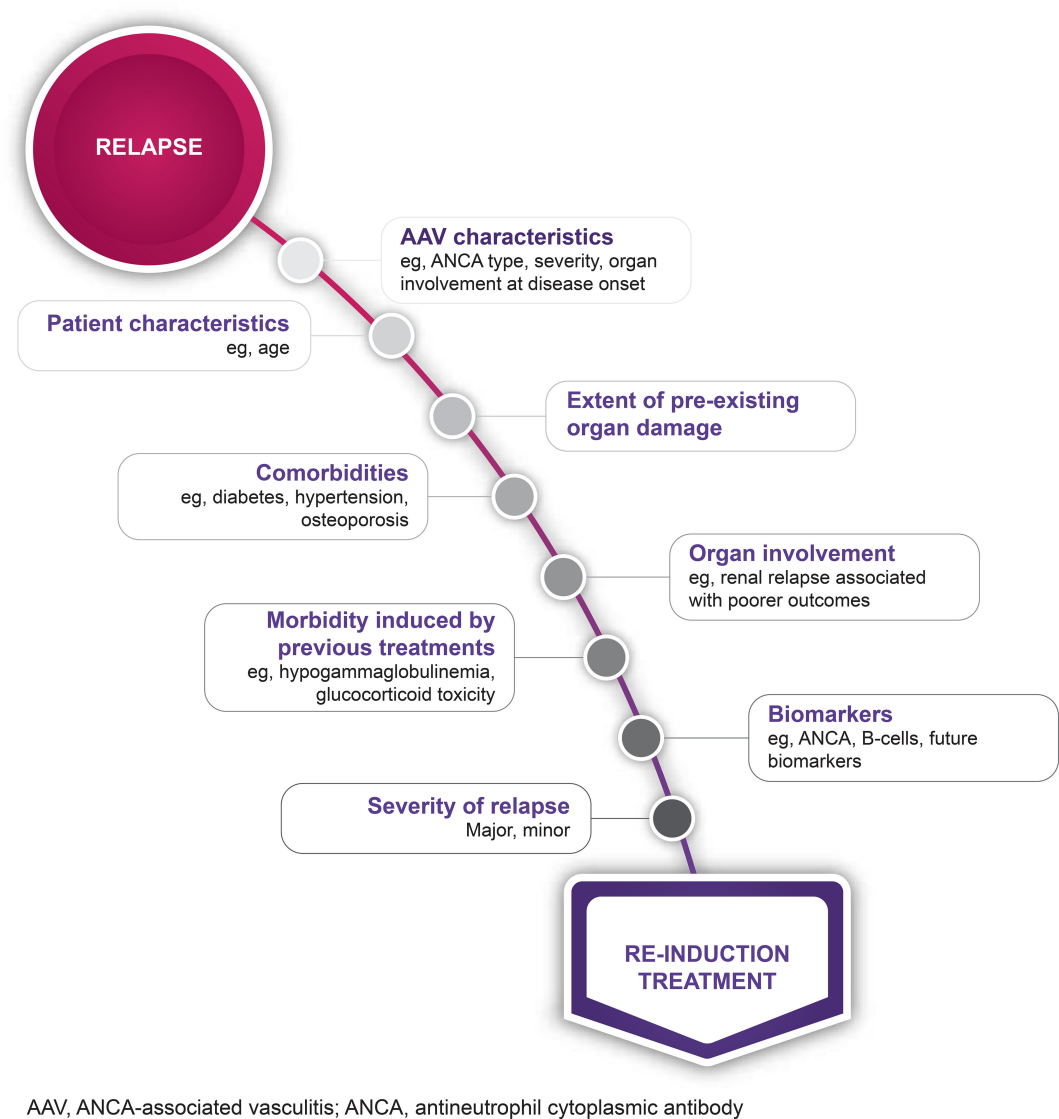
Considerations when choosing a re-induction treatment strategy. AAV, antineutrophil cytoplasmic antibody- (ANCA-) associated vasculitis.

There is no “one-size-fits-all” solution, and decisions regarding re-induction treatment should be based on an individual assessment considering ANCA type, disease severity, comorbidities, affected organs, and other patient characteristics such as age (**Figure 3**). A major relapse requires re-initiation of induction treatment with rituximab or cyclophosphamide, as employed to achieve remission in newly diagnosed cases (14, 33) (**Figure 2**). In contrast, a minor relapse typically warrants only temporary treatment intensification, such as a short course of low-dose glucocorticoids (**Figure 2**). It should be noted, however, that minor relapses are often a prelude to a more serious event, especially if there is an accompanying rise in ANCA titer (**Figure 2**). Among the 44 patients who experienced a minor relapse during follow-up in the RAVE trial, 80% achieved remission with an increase in prednisone dose, but 70% relapsed within 6 months (27). Patients who were least likely to maintain prolonged remission tended to have GPA, be PR3-ANCA positive and have previous relapses. This suggests that adjusting the dose and/or duration of immunosuppression according to patient characteristics might help prevent the need for more intensive treatment in the future. In patients with frequent relapses, alternative strategies beyond a temporary increase in glucocorticoids are recommended (14). This often involves combining a low-dose glucocorticoid with an immunosuppressive agent, most commonly rituximab. However, as previously discussed, emerging evidence suggests that glucocorticoids might not be beneficial for all patients (81).

Although data specifically pertaining to the treatment of relapse are scarce, many studies have included patients with a history of relapse (**Table 1**). Treatment of relapse typically includes rituximab and/or cyclophosphamide in addition to glucocorticoids (**Figure 2**). Rituximab has been shown to be as effective as cyclophosphamide in achieving remission and is more effective than azathioprine for maintaining remission (8, 37, 48, 107). A retrospective analysis of data from the RAVE trial found that 88% of patients with a major relapse six to 18 months after initial induction therapy achieved remission following re-induction with rituximab and glucocorticoids (47). Similarly, the RITAZAREM trial found that 90% of patients relapsing with GPA or MPA achieved remission within four months of starting re-induction therapy with rituximab and glucocorticoids (51). However, there is a minority of patients for whom AAV remission is not induced by rituximab (51). Reasons for this are unclear, but a single nucleotide polymorphism of the B-cell activating factor (BAFF) gene and high interpatient variability of serum rituximab levels have been proposed as potential causes (55, 56). Furthermore, data from the RITAZAREM trial confirmed findings from previous retrospective studies, in which higher glucocorticoid doses were associated with an increased risk of developing low IgG levels (48, 74). This supports the use of glucocorticoid-sparing treatment regimens for reducing the risk of infection.

Avacopan effectively enables a reduction in glucocorticoid exposure and glucocorticoid-related toxicity in patients receiving rituximab or cyclophosphamide for relapsing AAV (**Table 1**). The ADVOCATE trial demonstrated that avacopan was as effective as a prednisone taper as induction therapy in patients with new-onset or relapsing AAV treated with rituximab or cyclophosphamide, with avacopan-based treatment demonstrating a 54% estimated reduction in relapse risk compared to a prednisone taper (40). A supplementary analysis confirmed that avacopan effectively achieved remission in the subgroup of patients with relapsing disease (40). Importantly, patients who received the avacopan-based regimen received only a third of the cumulative dose of glucocorticoids (equating to a median of 2100 mg less total prednisone-equivalent over a year) compared with the prednisone taper group and experienced statistically significant and clinically meaningful improvements in health-related quality of life (40, 108). Results from the subset of patients who received rituximab echoed the main findings of the ADVOCATE trial, showing a 58% reduction in relapse risk in the avacopan group compared with prednisone (109).

Another benefit for avacopan is that it provides greater kidney function recovery than prednisone taper (40, 110, 111). The least-squares mean improvement in eGFR from baseline for patients with kidney involvement in the ADVOCATE study was significantly greater for those in the avacopan group than for those in the prednisone taper group at 52 weeks (7.3 vs 4.0 mL/min/1.73 m^2^; p=0.0259) (40, 112). An even greater improvement from baseline to week 52 (16.1 vs 7.7 mL/min/1.73 m^2^; p=0.003) was seen among patients with impaired kidney function at baseline (eGFR <20 mL/min/1.73 m^2^) (111). This is particularly relevant for patients requiring re-induction because (as previously discussed) each relapse increases the risk of organ damage due to increased inflammatory disease activity and the use of higher-dose (more toxic) re-induction treatments (31).

Evaluation of avacopan use in clinical practice, including patient categories not eligible for inclusion in the ADVOCATE trial, confirmed the high response rates and steroid-sparing effect of the avacopan regimen (113–115). Furthermore, a small study of Italian clinical practice revealed that avacopan was largely used in relapsing disease and was associated with a high sustained remission rate (116).

**Figure 4:**
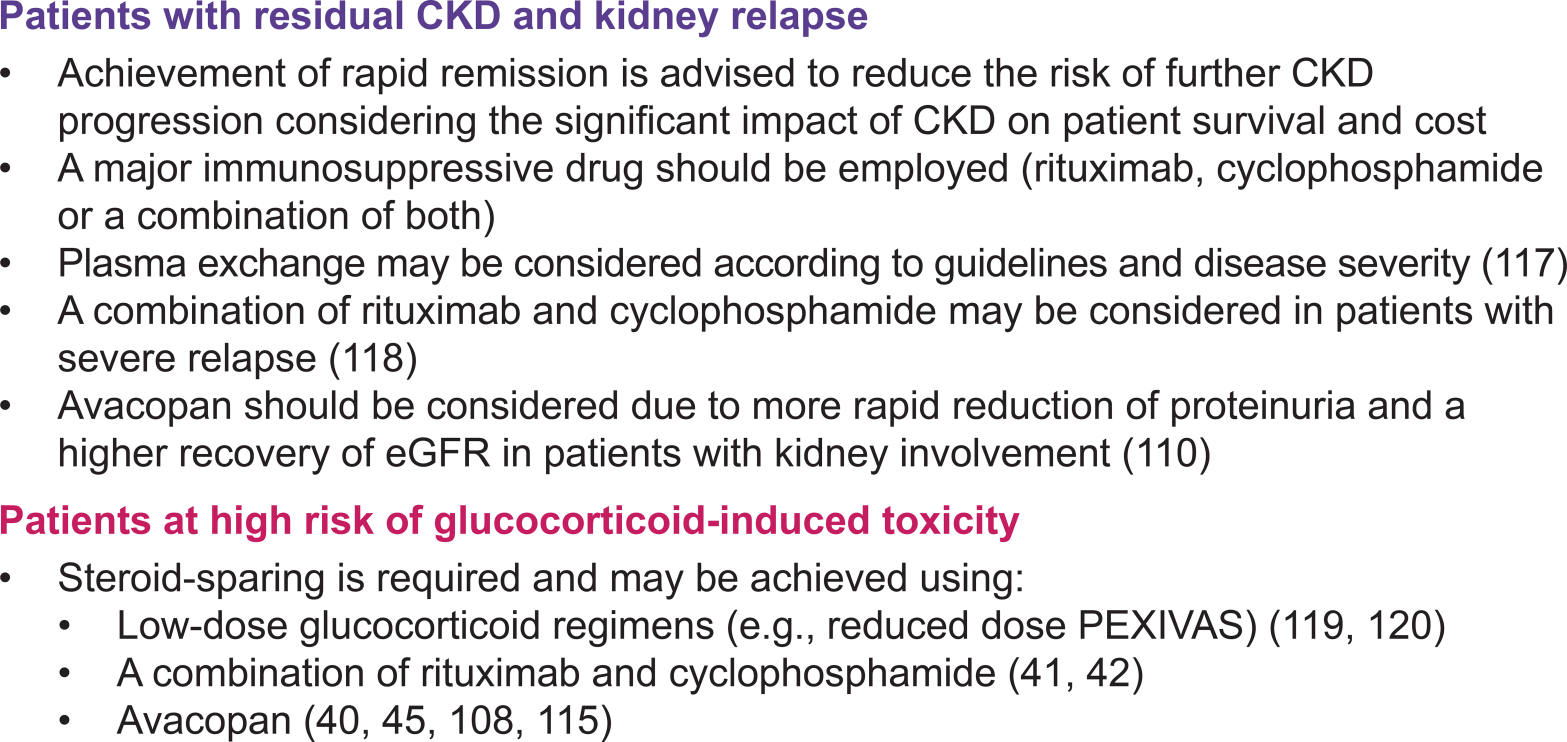
Scenarios illustrating wider treatment options in the management of AAV. AAV, antineutrophil cytoplasmic antibody- (ANCA-) associated vasculitis; CKD, chronic kidney disease; eGFR, estimated glomerular filtration rate.

Overall, evidence supports the use of rituximab in patients at risk of relapse, and the inclusion of avacopan in the induction regimen, at least in patients who would otherwise be treated with prednisone and in those requiring full re-induction. As the therapeutic armamentarium expands, greater emphasis must be placed on personalizing treatment to achieve effective disease control while minimizing treatment-related toxicities (**Figure 4**).

## 7 Impact of patient perspectives on treatment decisions

Health-related quality of life (HR-QoL) is often lower in patients with AAV compared to the general population, with many patients reporting depression, anxiety, unemployment, fatigue and pain (26, 108, 121). As expected, patient-reported outcomes (PRO) tend to worsen during periods of active disease and improve with remission (121). A *post-hoc* analysis of PRO data from the ADVOCATE trial demonstrated improvements in HR-QoL from baseline to weeks 26 and 52 in patients with AAV treated with rituximab or cyclophosphamide alongside glucocorticoids (108). Improvements in 36-Item Short Form Health Survey (SF-36) summary scores were greater in patients receiving avacopan versus prednisone taper at weeks 26 and 52, while EuroQoL 5-Dimensions 5-Level Questionnaire (EQ-5D-5L) summary scores showed greater improvements for avacopan versus prednisone taper only at week 52 (108). Whether this is directly due to the effects of avacopan, the impact of lower cumulative glucocorticoid exposure, or a combination of both remains to be established in future studies. Either way, results suggest that patient-reported HR-QoL should be taken into account when choosing a treatment regimen.

EULAR guidelines for the treatment of AAV stress the importance of effective communication and shared decision-making between physicians and patients (14). However, results from an online survey completed by 170 healthcare professionals (HCP) and 69 patients suggest that HCPs and patients have different perspectives on treatment aims, with HCPs prioritizing treatment efficacy and clinical outcomes, while patients focus more on long-term outlook, mental health, and functional impact (122). Patient perspectives may also be shaped by individual experiences and disease history. For example, a survey of 470 patients with AAV from 13 countries found that patients with prior exposure to dialysis or plasma exchange were more likely to favor plasma exchange for relapse management than patients without prior dialysis or plasma exchange (123). This highlights the need for a more patient-centered approach to AAV management.

## 8 The way forward

Future studies are required to evaluate optimal dosing strategies and new combinations of immunosuppressants in patients with AAV. Ongoing studies include the COMBIVAS trial, in which patients with AAV are treated with rituximab in combination with the anti-BAFF agent, belimumab (55, 124, 125). The rationale for this study is that the increased BAFF levels reported in some patients with AAV may hinder the efficacy of rituximab. It is hoped that the distinct mechanisms of action for rituximab and belimumab will enhance B-cell targeting, especially as belimumab appears to mobilize CD27+ memory cells making them available to the cytotoxic effect of rituximab (126). Similarly, since activated CD4 T-cells are involved in the pathogenesis of AAV and are likely central in mediating kidney damage, a therapy that inhibits T-cell activation could help control disease activity (127, 128). However, the ABROGATE trial did not demonstrate reduced relapse rates in patients with GPA treated with abatacept (129). Data from patients with other indications suggest that obinutuzumab may achieve greater tissue B-cell depletion than rituximab (130). The OBIVAS trial is comparing the effects of obinutuzumab and rituximab on B-cell depletion and sustained remission in patients with AAV, with completion expected in late 2025 (131). Over time, the options for re-induction are likely to expand enabling further reductions in glucocorticoid use.

## 9 Concluding remarks

Effective relapse prevention and/or treatment is critical to minimizing tissue damage and glucocorticoid exposure in patients with AAV. Treatment choices are not straightforward due to interpatient variability in response and the high proportion of patients with significant comorbidities, many of which may be AAV-induced or treatment related. Balancing the benefits of treatment against individual risk factors, treatment toxicities, and organ damage is key to optimizing outcomes.

There is no definitive biomarker for predicting AAV relapse; however, monitoring ANCA titers and (in rituximab-treated patients) B-cell repopulation may help identify patients with a high risk of relapse, thereby guiding preventative dosing. When relapse prevention fails, early treatment re-introduction or intensification will help minimize the effects of active disease on tissue damage and optimize long-term outcomes. Rituximab effectively maintains remission and re-induces remission after relapse. Adjunctive therapy with avacopan can help minimize glucocorticoid exposure while improving kidney function and achieving lower relapse rates than a prednisone-based regimen aimed at corticosteroid withdrawal (40).

Further studies are required to investigate the effects of re-induction treatments specifically in patients with AAV relapse. Although specific data are lacking, it is likely that the pathogenetic mechanisms underlying new-onset AAV and AAV relapse are similar. Importantly, clinical evidence to date supports the comparable efficacy of the same therapeutic approaches in both settings. In both scenarios, reducing the risk of organ damage by minimizing disease activity whilst limiting treatment toxicity remains a top priority.
